# Habitat orientation alters the outcome of interspecific competition: A microcosm study with zooplankton grazers

**DOI:** 10.1002/ece3.3909

**Published:** 2018-02-19

**Authors:** Ying Pan, Yunshu Zhang, Shucun Sun

**Affiliations:** ^1^ Yunnan Key Laboratory for Plateau Mountain Ecology and Restoration of Degraded Environments School of Ecology and Environmental Sciences Yunnan University Kunming China; ^2^ Department of Ecology School of Life Sciences Nanjing University Nanjing China; ^3^ ECORES Lab Chengdu Institute of Biology Chinese Academy of Sciences Chengdu China

**Keywords:** cladoceran species, habitat orientation, interspecific competition, phytoplankton–zooplankton dynamics, spatial property, species coexistence

## Abstract

Habitat orientation has recently been demonstrated to affect the foraging behavior, growth, and production of plankton grazers. Because the orientation effect may vary with species, we hypothesize that habitat orientation may alter interspecific interactions between animal species. We experimentally investigated how habitat orientation (placing cuboid chambers in three orientations with long, medium, and small side as the chamber height) affected the interaction between two common cladoceran species, *Daphnia magna* and *Moina micrura*, which competitively exploited green algae of *Chlorella pyrenoidosa* at two volume scales (64 and 512 ml). Results show that chamber orientation and volume additively affected the behavior and species performance of the grazers. Specifically, both grazer species generally decreased their average swimming velocity, grazing rate (on algal cells), body size, and survival and reproduction rates with increasing chamber height for both chamber volumes and with decreasing chamber volume regardless of chamber orientation. Nevertheless, the decrease magnitude was greater for *M. micrura* with increasing chamber height but was greater for *D. magna* with decreasing chamber volume. Correspondingly, when cocultured, the density ratio of *D. magna* to *M. micrura* increased with increasing chamber height but decreased with decreasing chamber volume. At the end of the experiment, none of *D. magna* individuals survived in the small and short (large‐based) chambers, and few *M. micrura* individuals survived in large and tall (small‐based) chambers. These results indicate that both habitat orientation and size affect the outcome of interspecific competition between grazer species. We suggest that variation in habitat orientation may improve community coexistence and species diversity in nature.

## 1 INTRODUCTION

Spatial property is one of the factors affecting species interaction and diversity (Whalen, Aquilino, & Stachowicz, 2016; Yeager, Keller, Burns, Pool, & Fodrie, 2016). For example, different species often perform best in different habitat spaces as the optimal requirement for spatial conditions differs among species, resulting in high species diversity in heterogeneous habitats (Amarasekare, 2003; Mcclain & Barry, 2010). Studies have substantially shown that habitat size (Cheruvelil, Soranno, Webster, & Bremigan, 2013; Martínez‐Jerónimo, Espinosa‐Chávez, & Villaseñor, 2015; Westphal, SteVan‐Dewenter, & Tscharntke, 2006), shape (Pan, Zhang, Peng, Zhao, & Sun, 2015; Wakano, Ikeda, Miki, & Mimura, 2011), and connectivity (Baggio, Salau, Janssen, & Schoon, 2011; Karsai & Kampis, 2011) significantly affect the outcome of interspecific competition and hence species coexistence. However, one another spatial feature of habitats, habitat orientation, has rarely been studied in relation to species interaction and coexistence (Pan & Sun, 2016).

Even for habitats having the same size and shape, they may differ in how they are placed in space; this geometric feature is so‐called habitat orientation (Pan & Sun, 2016). Habitat orientation is diverse in nature and can have ecological consequences at different organization levels. For example, narrow terrestrial forest patches could be placed either perpendicular or parallel to the migratory direction of bird species, which have differential effects on bird abundance (Hollenbeck & Ripple, 2007). Similarly, narrow aquatic plant patches could be situated perpendicular or parallel to water current (Tanner, 2003), harboring different levels of fish abundance and diversity. Recent studies provide direct experimental evidence that habitat orientation affects the foraging rate (Pan & Sun, 2016), survival, growth, and reproduction, as well as population dynamics of zooplankton grazers (Zhang, Pan, Chen, Hu, & Sun, 2017), showing its ecological consequences at the individual and population level.

**Figure 1:**
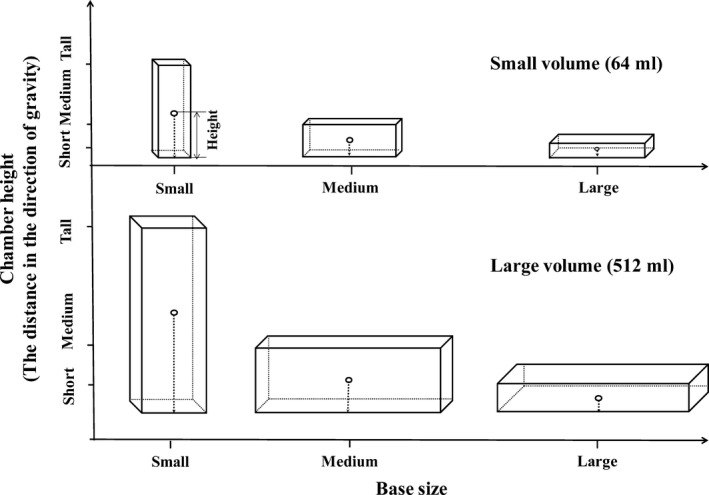
Illustration showing three levels of habitat orientation and two levels of chamber volume. The side length ratio was 1:2:4 for each chamber

Here, we further propose that habitat orientation may exert species‐specific effects on species performance and hence affect interspecific competition of aquatic organisms. Taking cuboid chambers for phytoplankton–zooplankton systems as an example, if the chamber volume and the ratio of length: width: height are constant, varying habitat orientation will change vertical length (in the direction of gravity), which is longer in tall (small‐based) chambers than those in short (large‐based) ones (see Figure 1). Thus, tall chambers can lead to higher proportion of vertical movement compared to short chambers for zooplankton grazers (e.g., cladoceran species) due to the random walk strategy of these organisms in aquatic environments (Visser & Thygesen, 2003). Given that cladoceran grazers generally have to spend more energy to swim upward the same distance than individuals swimming horizontally as a result of additional costs of overcoming gravity, swimming velocity of these grazers should be lower in tall chambers than in short ones (Pan et al., 2015).

Such behavioral responses of cladocerans to habitat orientation would transmit to affect their grazing rate and thus body growth, and survival and reproduction rates due to the significant positive relationships between swimming velocity and grazing rate (Christensen, Lauridsen, Ravn, & Bayley, 2005; Visser & Kiørboe, 2006) and between grazing rate with body growth, and survival and reproduction rates (Rinke & Vijverberg, 2005; Zhang et al., 2017).

Importantly, cladoceran grazers may have differential responses to habitat orientation among species that coexist in the same aquatic community. For example, body shape has a greater influence in determination of the drag and energy consumption of aquatic organisms when they moving through water. As a result, cladocerans (e.g., *Daphnia* sp.) with higher ratio of body depth to width should be more appropriate to swimming vertically than horizontally (Weber & Noordwijk, 2002; Zhang et al., 2017) and thus would be more adapted to narrow and deep waters; while in wide and shallow habitats, the species with lower ratios of body depth to body width should be better adapted. If the interspecific difference in the responses due to body shape is large enough, the habitat orientation would possibly affect the outcome of the interspecific competition.

We in this study determined the effects of habitat orientation (three levels: placing cuboid chambers in three orientations with long, medium, and small side as the chamber height, respectively) on interspecific competition between two cladoceran species *Daphnia magna* and *Moina micrura*, which were fed with the chlorophyte *Chlorella pyrenoidosa* at two chamber volumes (64 and 512 ml). We collected the data on the velocities of horizontal swimming, upward swimming. and downward swimming, and the time allocation among horizontal swimming, upward swimming, downward swimming, and quiescence, as well as species performance (e.g., grazing rate, body size, and survival and reproduction rates), and then, we determined the relative competitive ability between *D. magna* and *M. micrura*. We predicted that (1) *D. magna* would be more appropriate to swimming and grazing in tall chambers than short ones (because it had a higher ratio of body depth to width compared to *M. micrura*), and (2) thus, the survival, growth and reproduction rates, as well as the competitive ability of *D. magna*, would be higher in the tall chambers.

## 2 MATERIALS AND METHODS

### 2.1 Experimental organisms

Our experimental organisms included two cladoceran species (*D. magna* and *M. micrura*) and one algal species (*Chlorella pyrenoidosa*). *D. magna* and *M. micrura* were collected from Lake Taihu (31.5°N, 120.1°E), East China. These two cladoceran species are reported to coexist in many freshwater ecosystems worldwide, such as lakes, reservoirs, rivers, and ponds (Chen et al., 2016; Marcé, Comerma, García, Gomà, & Armengol, 2005; Zhao, Sun, Huang, & Dai, 1996). Moreover, *D. magna* adults are much larger (adult size 2.3–6.0 mm vs. 0.5–1.3 mm) and seem to prefer vertical movement by spending more time on vertical movement than *M. micrura* adults (Pan et al., 2015). Green alga *C. pyrenoidosa* (FACHB‐28) was obtained from the Freshwater Algae Culture Collection of the Institute of Hydrobiology, the Chinese Academy of Sciences. The two cladoceran species were fed with *C. pyrenoidosa* at a rate of 10^5^ cells/ml per day for more than 3 months prior to the experiments. Both grazer and algal species were grown in COMBO in an incubator at 20 ± 0.5°C with a 14‐hr light (at 50 μmol photons m^−2^ s^−1^): 10‐h dark cycle.

Prior to the experiments, the grazers (about 2–3 days old) were transferred to clean COMBO medium and starved for at least 4 hr to increase motivation to forage. Only medium‐sized individuals of each grazer species (1.10 ± 0.09 mm long for *D. magna* and 0.83 ± 0.07 mm long for *M. micrura*; the data denote mean ± *SD*,* n *=* *12) were used as experimental materials because large individuals might bear embryos and reproduce during short‐time experiments and small individuals were hard to trace during behavioral observations. Reynolds numbers of *D. magna* and *M. micrura* were always greater than 2.5 according to empirical formulas: Reynolds numbers = *UL*/*v*, where *U* is the three‐dimensional instantaneous velocity (ranging from 0.26 to 0.46 cm/s and from 0.33 to 0.56 cm/s for *D. magna* and *M. micrura*, respectively; calculated from the results of this study); *L* is the body length (cm); and *v* is the coefficient of kinematic viscosity (cm^2^/s, Svetlichny & Hubareva, 2005).

### 2.2 Experimental design

The experiment was designed to include six chamber treatments, that is, three levels of habitat orientation (varied the orientation of cuboid chambers with the small, medium, and large side as base, respectively) × two levels of chamber volume (64 and 512 ml), with each treatment having 18 cuboid chambers (transparent polyethylene containers, see Figure 1). The side length ratio was 1:2:4 for each chamber. The 18 cuboid chambers (for each treatment) were used for three different (sub) experiments on grazer behavior, grazing rate, and interspecies competition, with each experiment having six replicate chambers. Each chamber was set as a cocultured plankton system, in which cocultured *D. magna* and *M. micrura* were grazers and the alga *C. pyrenoidosa* was their exclusive diet. The grazer activity and grazing rate were measured 4 hr after the start of the experiment. However, the competition experiment lasted for 32 days. Furthermore, in both grazing rate and competition subexperiments, we set up an additional treatment that included the algal species only, such that the grazing rate of the cocultured grazer species and the population dynamics of the algal species in the absence of grazers could be estimated. Each of such additional treatments had six replicates.

Prior to the experiment, each experimental chamber was gently aerated with sterile filtered air (Sartorius, Midisart 2000) and mechanically stirred (at 90 rpm using an incubator) for 10 min to homogenize dissolve oxygen (DO) concentration and algal density between chambers of different treatments. Then, grazers were transferred into the chambers that were designed to include grazers with the density of 125 individuals/L (half *D. magna* and half *M. micrura*), followed by the introduction of concentrated inoculums of *C. pyrenoidosa*. The grazer density is within the range of previous studies addressing species interactions and cladoceran behavior (from 50 individuals/L in Pan et al., 2017, 300 individuals/L in Hurtado‐Bocanegra, Nandini, & Sarma, 2002; to 400 individuals/L in Lürling, 2003). The algal density was set relatively low (2.5 × 10^4^ cells/ml) to minimize the reaction of positive phototaxis, but it was still above the incipient limiting level for grazer species and has been used by many other microcosm experiments (e.g., Gilbert, 1990; Pan, Zhang, & Sun, 2014).

All the chambers were capped with breathable polyethylene films and then transferred to incubators (TS‐2102GZ, Shanghai Anjing laboratory equipment Co., Ltd) that were set at 20 ± 0.5°C with light intensity being 50 μmol photons m^−2^ s^−1^. They were mechanically stirred (at 90 rpm) and gently aerated with sterile filtered air (Sartorius, Midisart 2000) for 5 min every 1 hr throughout the experiment to facilitate gas exchange (to homogenize DO concentration between treatments) and keep the algae in suspension. Measurements indicated that algal density was indistinguishable between the top and bottom layers of the experimental chambers an hour after a stirring event (see Appendix 1).

### 2.3 Behavior experiment

Behavior experiment was carried out in a laboratory at 20 ± 1°C, which was achieved by an air conditioner. Cool‐white fluorescent bulbs were installed approximately 2 m away around (including the top and all sides) the experimental setup providing illumination (at 40 μmol photons m^−2^ s^−1^) to homogeneous light condition following our previous studies (Pan et al., 2015). Such a physical setting may not only minimize the grazer reaction of positive phototaxis that confounds the random walk pattern of zooplankton grazers (Garcia, Moss, Nihongi, & Strickler, 2007; Komin, Erdmann, & Schimansky‐Geier, 2004), but also allow for our results applicable to the animals that do not show phototaxis in aquatic systems (Zhang et al., 2017).

Swimming activities of grazer individuals were recorded 4 hr after the beginning of the experiment with two video cameras (1,920 × 1,080 pixels), which were placed orthogonally at a distance of 26 cm from the centre of the projective plane (i.e., the bottom or the backside of the chamber). This physical setting allowed for concurrently recording the swimming behavior of a zooplankton individual in both horizontal and vertical directions. After 15 min of acclimation, the recording began and finished after 5 min of filming at 60 frame/s.

Video recordings were transferred to computer, and four types of behaviors were recorded using an image measurement tool (Adobe After Effects CS4) for the grazers, that is, horizontal swimming, upward swimming (swim vertically upward), downward swimming (swim directly downward), and quiescent status. Firstly, we recorded the time for the four types of behaviors. Then, we chose the fragments that contained swimming trajectories away from the chamber walls (in the middle of both views of the camera) and were longer than 2 s (following Gorski & Dodson, 1996; Moison, Schmitt, & Souissi, 2012) to calculated instantaneous swimming velocity as the distance swum by the grazer individuals between two frames (i.e., 16.9 ms) using ImageJ 1.46 and MTrackJ plugin (Artells et al., 2013; Manenti, Denoël, & Ficetola, 2013). Each video was first calibrated to convert pixels into real distances (mm) using reference marks when analyzing the instantaneous swimming velocity. Finally, the average swimming velocity (*V*_average_, mm/s) was approximated according to the formula that was used in our previous studies (Pan & Sun, 2016; Pan et al., 2015): *V*_average_ **= (*V*_*h*_ *T*_*h*_ **+ *V*_*u*_ *T*_*u*_ **+* V*_*d*_ *T*_*d*_)/(*T*_*h*_ + *T*_*u*_ +* T*_*d*_ **+ *T*_*q*_), where *T*_*h*_, *T*_*u*_, *T*_*d,*_ and *T*_*q*_ were the durations of horizontal swimming, upward swimming, downward swimming, and quiescence (s), respectively; *V*_*h*_, *V*_*u*_, and *V*_*d*_ were the velocities of horizontal, upward, and downward swimming (mm/s). Three individuals of each grazer species were randomly chosen to determine the above‐mentioned metrics in each chamber, and thus, a total of 108 individuals per grazer species (3 individuals per chamber × 6 chambers per treatment × 6 treatment) were followed. All these metrics of each grazer obtained during the behavioral investigation was averaged for each chamber before data analyses. We additionally calculated swimming velocity ratio as the ratio of average swimming velocity of *D. magna* to *M. micrura*.

### 2.4 Grazing experiment

Investigation of grazing rate (i.e., clearance rate) was conducted in the incubators (TS‐2102GZ, Shanghai Anjing laboratory equipment Co., Ltd) that were set at 20 ± 0.5°C with light intensity being 50 μmol photons m^−2^ s^−1^. The algae were sampled 4 hr after the beginning of the experiment. During sampling, we first measured the concentration of DO using a Hach HQ40d oxygen probe (Hach, Loveland, Colorado, USA) as soon as the chambers were taken out of the incubator. Results showed that DO was unaffected by habitat orientation and chamber volume and was always higher than 6.7 mg/L (see Appendix 2), indicating that oxygen availability was not a potential limitation for the growth of grazer individuals after an intermittent stirring event. Then, 2 ml of the sampled solution was removed to a 10 ml tube that contained 0.1 ml of Lugol's preservative for microscopic enumeration of algal cells. At the end of the experiment, algal density was determined using a light microscope at 400× magnification.

The grazing rate (*G*, ml animal individual^−1^ hr^−1^) was calculated as the difference in algal density between the experimental treatments (with grazer) and the corresponding controls (without grazers) following the formula of Pace, Porter, and Feig (1983): *G *= *V *× [In (*C*_0_/*C*_1_)]/*NT*, where *V* is the chamber volume (ml); *C*_0_ and *C*_1_ are the algae density at the end of the measurement in the control and experimental chambers (cell/ml), respectively; *N* is the number of the grazer individuals within the chamber; and *T* is the duration of the experiment (4 hr).

### 2.5 Competition experiment

This experiment was designed to evaluate whether habitat orientation affected interspecific competition between the two grazer species after long‐term cocultures (32 days). The experiment was carried out in incubators that were controlled under the same conditions as for the investigation of grazing rate, except for using a light–dark cycle of 14 hr:10 hr. Microcosms were sampled on days 2, 4, 6, 8, 10, 12, 14, 17, 20, 24, 28, and 32 at 8:00 after the treatments were initiated. The number of individuals was counted for each grazer species in each chamber before each sampling. During sampling, 5% of the volume was removed from each chamber, and 2 ml of these removed solutions was transferred into a 10 ml tube that contained Lugol's preservative for the measurement of *C. pyrenoidosa*. Then, 5% volume of fresh medium was added to each chamber to replenish nutrients and prevent metabolic waste buildup. Subsequently, each chamber was capped and replaced back into the incubator.

Additionally, body size, reproduction, and survival rates of *D. magna* and *M. micrura* were estimated on day 6 when the size differences between the parental and neonatal grazers were most obvious and when the first‐generation offspring of the grazers had not begun producing new neonates according to previous studies (e.g., Pan et al., 2014; Zhang et al., 2017). Body size of each grazer species was determined using all (<5) survived adult females in small chambers and five randomly chosen adult females in large chambers following Zhang et al. (2017) as the length from top of the head to tip of the abdomen, which was measured using an inverted light microscope at ×40 magnification. Body size was averaged for each chamber before data analyses because the number of grazer individuals differed largely among chambers. Reproduction rate (*No/Ni*, %) and survival rate (*Ns/Ni, %*) were estimated as the number of offspring (*No*) and survived adults (*Ns*) to the total number of individuals added to the chamber at the initial of experiment (*Ni*), respectively. Body size ratio and survival rate ratio were defined as the ratio of body size and survival rate of *D. magna* to *M. micrura*, respectively.

### 2.6 Data analysis

All data were tested for normality and variance homogeneity prior to analyses. Data on swimming velocities were log‐transformed to achieve the normality and homogeneity. Two‐way ANOVAs were used to determine the effect of habitat orientation and chamber volume on swimming behavior, average swimming velocity, and grazing rate, density, and other species performance variables (including body size, reproduction, and survival rates, as well as the swimming velocity ratio, body size ratio, survival rate ratio, and density ratio of *D. magna* to *M. micrura*) for both grazer species and the cell density of the algal species on a specific day, followed by the Tukey test for multiple comparisons once a significant effect was detected. Three‐way ANOVAs were used to determine the effects of habitat orientation, chamber volume, and grazer species on average swimming velocity. Two‐way repeated measures ANOVA (RM‐ANOVA) were used to test the effects of treatment factors (habitat orientation and chamber volume) on algal cell, grazer density, and density ratio of *D. magna* to *M. micrura* during the competition experiment, followed by the Tukey test for multiple comparisons once a significant effect was detected. The sphericity assumption was evaluated with the Mauchly's test, and in the case of violation, the Greenhouse–Geisser correction was applied to recalculate the *F*‐value. In addition, linear regression analyses were conducted to determine the relationships among treatment factors mentioned above. All the analyses were carried out using IBM SPSS19.0 package (SPSS Inc., USA).

## 3 RESULTS

### 3.1 Swimming activity

**Figure 2:**
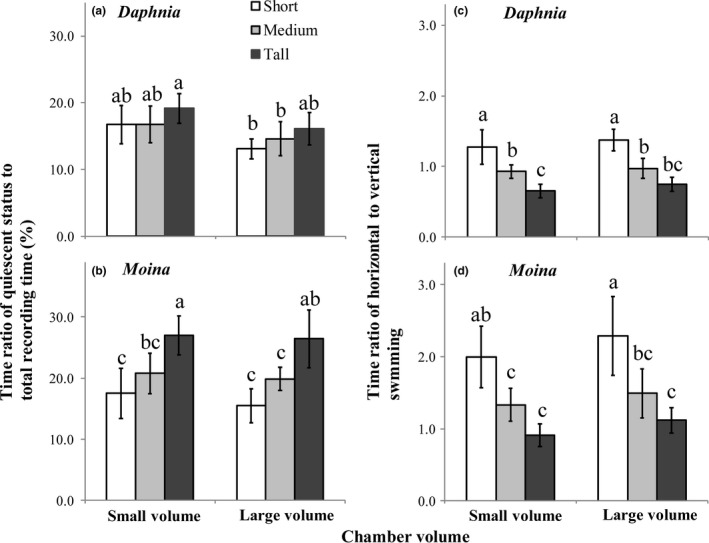
Time ratios (means ± 1 *SD*,* n* = 6) of quiescent status to total recording time (a, b) and of horizontal to vertical swimming (c, d) of grazer species *Daphnia magna* and *Moina micrura* when they were cultured in combination with the green alga *Chlorella pyrenoidosa* as the exclusive diet under three levels of habitat orientation and two levels of chamber volume. Different letters indicate significant differences among treatments. Multiple comparisons of treatment means were performed using Tukey test at the 0.05 significance level

**Figure 3:**
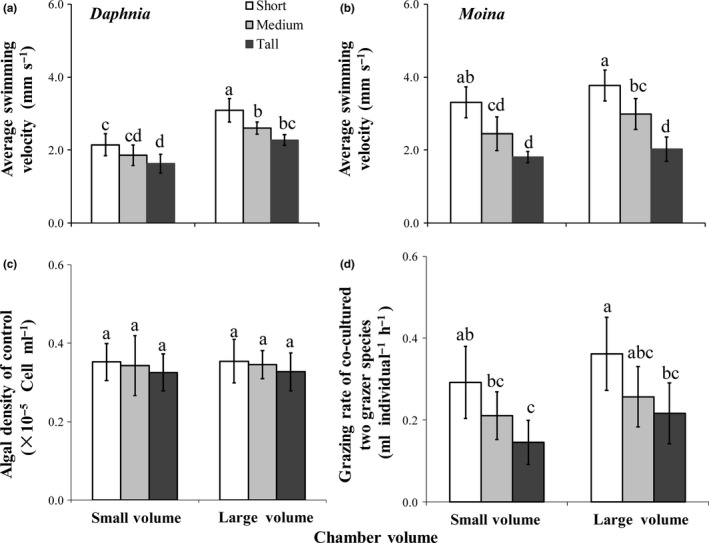
Average swimming velocities (means ± 1 *SD*,* n* = 6) of *Daphnia magna* (a) and *Moina micrura* (b) and their combined effects (d, means ± 1 *SD*,* n* = 6) in foraging algal *Chlorella pyrenoidosa* (using the treatment without predators as controls, c) when they were cultured in combination under three levels of habitat orientation and two levels of chamber volume. Different letters indicate significant differences among treatments. Multiple comparisons of treatment means were performed using Tukey test at the 0.05 significance level

Both chamber orientation and volume, but not their interaction, significantly affected swimming activity and average swimming velocity for both *D. magna* and *M. micrura* (Figures 2 and 3; Tables 1 and 2). For both chamber volumes, increasing chamber height generally led to increases in the durations of quiescence and upward swimming (Table 2), thereby decreasing the time ratio of horizontal to vertical swimming in both grazer species (Figure 2c,d). Nevertheless, increasing chamber height led to a decrease in both horizontal and upward swimming velocities (Table 2). Consequently, average swimming velocity decreased with increasing chamber height for both grazers species (Figure 3a,b). For all three orientations, increasing chamber volume generally led to increase in the duration of horizontal movement and both horizontal and upward swimming velocities. As a result, average swimming velocity was generally higher in large chambers than small ones for both grazer species (Figure 3a,b).

**Table 1:** Summary of ANOVA results (*F* and *p* values) showing the effects of habitat orientation, chamber volume, and/or grazer species on average swimming velocity and grazing rate (*n* = 6) in behavior and grazing experiments, respectively, under three levels of habitat orientation and two levels of spatial scales

	*df*	Average swimming velocity (mm/s)	Grazing rate (ml individual^−1^ hr^−1^)
Orientation (*O*)	2	74.903***	11.852***
volume (*V*)	1	59.793***	6.268*
Grazer species (*G*)	1	35.815***	
*O * × *V*	2	1.094^ns^	0.098^ns^
*O * × *G*	2	13.091***	
*V * × * G*	1	5.926*	
*O * × *V * × *G*	2	0.327^ns^	

^ns^ *p* > .05; *p < .05; **p < .01; ***p < .001.

**Table 2:** Swimming behavior (means ± *SD*,* n* = 6) of *Daphnia magna* and *Moina micrura* when they were cocultured under three levels of habitat orientation and two levels of chamber volume

		The durations of horizontal swimming, upward swimming, downward swimming, and quiescence (s)				The velocities of horizontal swimming, upward swimming, and downward swimming (mm/s)		
		Quiescent status	Horizontal move	Vertically ascent	Vertically descent	Horizontal move	Vertically ascent	Vertically descent
*D. magna*	SS	55.8 ± 9.9ab	154.0 ± 12.7ab	80.6 ± 8.4e	42.5 ± 8.8ab	2.7 ± 0.4bc	2.2 ± 0.3bc	2.7 ± 0.5a
	SM	57.0 ± 8.9ab	136.7 ± 12.2bc	101.9 ± 9.4cd	45.7 ± 6.3a	2.2 ± 0.5c	2.0 ± 0.4c	2.8 ± 0.7a
	ST	66.9 ± 7.8a	111.0 ± 7.8d	124.7 ± 12.4ab	46.8 ± 7.6a	2.0 ± 0.4c	1.7 ± 0.4c	2.7 ± 0.6a
	LS	44.4 ± 4.5b	170.5 ± 7.6a	86.2 ± 12.1de	38.5 ± 5.3ab	3.9 ± 0.5a	3.1 ± 0.6a	3.1 ± 0.1a
	LM	50.1 ± 8.2b	144.1 ± 14.3b	110.3 ± 9.9bc	39.1 ± 4.6ab	3.3 ± 0.4ab	2.7 ± 0.2ab	3.1 ± 0.6a
	LT	56.1 ± 8.7ab	124.4 ± 10.5cd	135.7 ± 11.1a	32.4 ± 2.9b	3.1 ± 0.4ab	2.2 ± 0.3bc	3.2 ± 0.7a
*M. micrura*	SS	58.9 ± 13.2bc	184.2 ± 19.8ab	58.0 ± 9.0c	35.9 ± 7.5a	4.4 ± 0.5ab	3.0 ± 0.4a	3.6 ± 1.3a
	SM	70.4 ± 10.9ab	152.8 ± 15.3cd	77.9 ± 9.0ab	38.2 ± 6.1a	3.3 ± 0.8bc	2.6 ± 0.5ab	3.4 ± 0.9a
	ST	91.2 ± 10.4a	116.9 ± 12.5e	89.2 ± 8.6a	41.1 ± 12.9a	2.6 ± 0.5c	2.1 ± 0.4b	2.8 ± 0.8a
	LS	52.0 ± 8.7c	195.6 ± 11.9a	57.0 ± 13.9c	31.9 ± 4.9a	5.0 ± 0.7a	3.1 ± 0.4a	3.5 ± 0.7a
	LM	67.4 ± 5.1bc	162.1 ± 19.5bc	66.7 ± 12.8bc	43.8 ± 6.5a	4.3 ± 0.7ab	2.9 ± 0.4a	2.9 ± 0.7a
	LT	90.6 ± 17.9a	132.1 ± 14.4de	74.5 ± 11.4abc	44.7 ± 7.8a	3.0 ± 0.6c	2.2 ± 0.2b	3.0 ± 0.6a

SS, small volume and short chamber height; SM, small volume and medium chamber height; ST, small volume and tall chamber height; LS, large volume and short chamber height; LM, large volume and medium chamber height; LT, large volume and tall chamber height.

Different letters indicate significant differences among treatments. Multiple comparisons of treatment means were performed using Tukey test at the 0.05 significance level.

However, the effects of chamber orientation and volume differed between grazer species (Tables 1 and 2). The negative effect of increasing chamber height on average swimming velocity was more prominent in *M. micrura* than in *D. magna*, while the positive effect of increasing chamber volume on average swimming velocity was stronger in *D. magna* than in *M. micrura*. Thus, swimming velocity ratio of *D. magna* to *M. micrura* was the highest in the large and tall chambers and was the lowest in the small and short chambers (Appendix 3).

### 3.2 Grazing rate

Both chamber orientation and volume significantly affected the algal density in the presence of grazers (as reflected by grazing rate) (Table 1) but not in the absence of grazers (Figure 3c). The grazing rate consistently decreased with increasing chamber height for both chamber volumes, and it also appeared to increase with increasing chamber volume for all chamber orientations (Figure 3d). Additionally, the grazing rate was significantly and positively associated with average swimming velocity of *D. magna* (*r* ^2^ = .767, *p *<* *.05; Appendix 4a) and *M. micrura* (*r* ^2^ = .923, *p *<* *.01; Appendix 4b), as well as the average swimming velocities (*r* ^2^ = .957, *p *<* *.01; Appendix 4c).

### 3.3 Species performance

**Figure 4:**
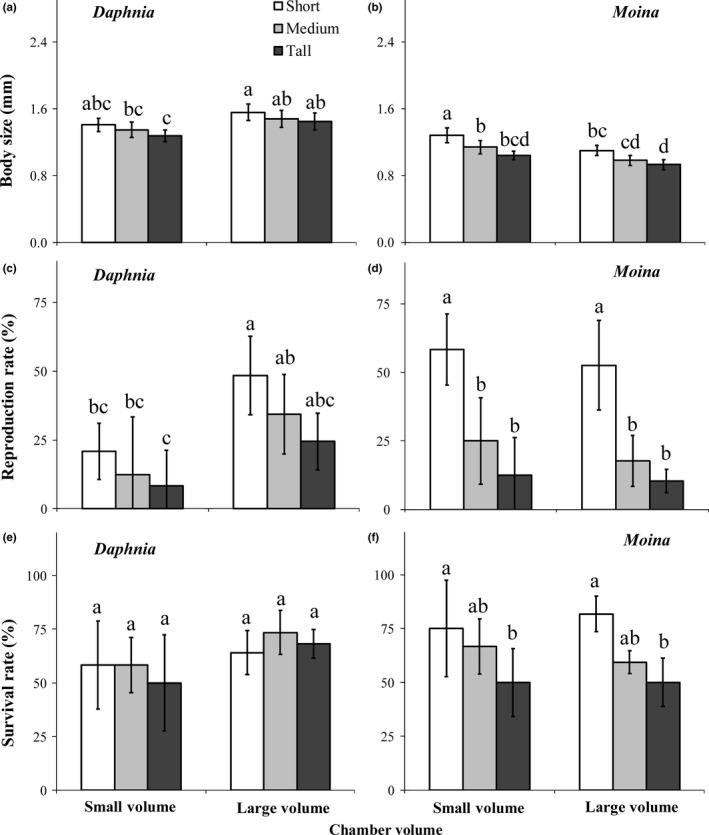
Variations (means ± 1 *SD*,* n* = 6) in body size (a and b), reproduction rate (c and d) and survival rate (e and f) of grazer species *Daphnia magna* and *Moina micrura* with green alga *Chlorella pyrenoidosa* as the exclusive diet when they were cocultured in the chamber on day 6 under three levels of habitat orientation and two levels of chamber volume. Different letters indicate significant differences among treatments. Multiple comparisons of treatment means were performed using Tukey test at the 0.05 significance level

Chamber orientation and volume also significantly affected the life history parameters of *D. magna* and *M. micrura* in the cocultured competition experiment (Figure 4). After 6 days of culture, the body size (Figure 4b), reproduction rate (Figure 4d), and survival rate (Figure 4f) of *M. micrura* consistently decreased with increasing chamber height for both chamber volumes. Moreover, the body size was smaller in the large chambers than small ones when the chamber bases were small or medium bases (Figure 4b). In the case of *D. magna*, the effects of habitat orientation on body size, reproduction rate, and survival rate were nonsignificant (Figure 4a,c,e). Nevertheless, for each orientation, body size (Figure 4a) and reproduction (Figure 4c) and survival rates (Figure 4e) of *D. magna* were generally greater in the large chamber than small ones.

**Figure 5:**
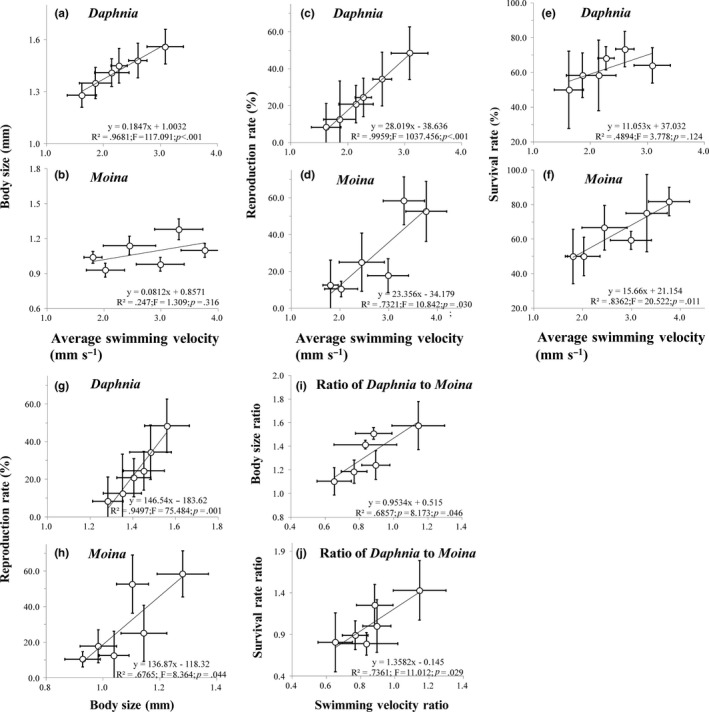
Linear regressions between average swimming velocity with body size (a and b), reproduction rate (c and d) and survival rate (e and f), and between body size with reproduction rate (g and h), of *Daphnia magna* and *Moina micrura* individuals, as well as between swimming velocity ratio with body length ratio (i) and survival rate ratio (j), of *Daphnia magna* to *Moina micrura*, respectively, under three levels of habitat orientation and two levels of chamber volume (*n* = 6)

Significant linear relationships were found between average swimming velocity and life history parameters in both grazer species (Figure 5a–f). Specifically, average swimming velocity was positively associated with body size for *D. magna* in both chamber volumes (Figure 5a) and for *M. micrura* in the large (*R* ^2^ = .999; *p = *.010) but not small chambers, with reproduction rate for both grazer species (Figure 5c,d) and with survival rate for *M. micrura* (Figure 5f) but not *D. magna*. Moreover, the body size was positively associated with reproduction rate for both *D. magna* and *M. micrura* (Figure 5g,h). Additionally, both body size ratio (Figure 5i) and survival rate ratio (Figure 5j) of *D. magna* to *M. micrura* were positively associated with swimming velocity ratio of *D. magna* to *M. micrura*.

### 3.4 Population dynamics

**Figure 6:**
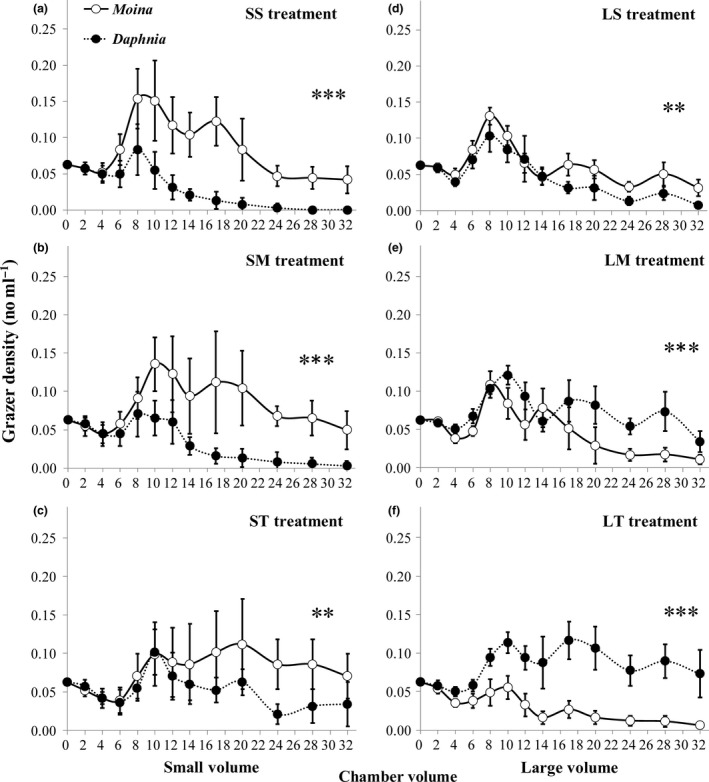
Densities (means ± 1 *SD*,* n* = 6) of *Daphnia magna* and *Moina micrura* when they were cocultured in the chamber throughout the month‐long experiment under three levels of habitat orientation and two levels of chamber volume. SS, small volume and short chamber height (a); SM, small volume and medium chamber height (b); ST, small volume and tall chamber height (c); LS, large volume and short chamber height (d); LM, large volume and medium chamber height (e); LT, large volume and tall chamber height (f). The symbol *, **, and *** indicate statistical significance at the .05, .01, and .001 level, respectively, between densities of the two grazers species through one‐way RM‐ANOVA

Chamber orientation and volume showed contrasting effects on population density between the two grazer species (Figure 6). Population density of *M. micrura* decreased, but population density of *D. magna* increased with increasing chamber height for both chamber volumes and with increasing chamber volume regardless of chamber orientation throughout the whole experiment. Thus, at the end of the experiment, *D. magna* was entirely excluded by *M. micrura* in the small and short chambers (Figure 6a), but *M. micrura* was almost totally exclude by *D. magna* in the large and tall chambers (Figure 6f).

Both chamber orientation and volume significantly affected the algal density in the presence of grazers (as reflected by grazing rate) but not in the absence of grazers throughout the experiment (Table 3). In the presence of grazers, despite the large variation in algal density with the experiment progressing, the algal density was generally lower in the short chambers than tall ones for both chamber volumes, and it was generally lower in the large chambers than small ones regardless of chamber orientation in the later stage of the experiment (Appendix 5).

**Table 3:** Results of two‐way repeated measures ANOVA (*F* and *p* values) showing the effects of time, orientation, and chamber volume on algal and grazer densities (*n* = 6) in different phytoplankton‐zooplankton systems

	Time (*T*)	Orientation (*O*)	Volume (*V*)	*T* × *O*	*T* × *V*	*O* × *V*	*T* × *O* × *V*
Cell density of control (cell/ml)	93.306***	1.571^ns^	2.158^ns^	1.106^ns^	0.571^ns^	0.177^ns^	0.261^ns^
Cell density of mixed culture	38.125***	72.682***	29.984***	1.501^ns^	3.695^ns^	1.970^ns^	0.667^ns^
Grazer density of cocultured *D. magna*	49.077***	55.933***	185.267***	9.130**	8.173**	8.461**	1.915^ns^
Grazer density of cocultured *M. micrura*	26.515***	24.194***	168.132***	3.542*	8.137**	7.321**	1.666^ns^
Density ratio of *D. magna* to *M. micrura*	6.936*	26.687***	59.595***	5.748*	12.302**	18.888***	5.626*

^ns^ *p* > 0.05; *p <* *0.05; **p <* *.01; ***p <* *.001.

## 4 DISCUSSION

The spatial competition theory that patchy environments may allow more species to coexist has achieved a great success in ecology (Mcclain & Barry, 2010; Tilman, 1994; Yeager et al., 2016). The present study is the first to show that habitat orientation affects the outcome of interspecific interaction between zooplankton grazers. Our results suggest that habitat orientation, in addition to size and shape, is an important abiotic factor that should be considered when trying to understand how spatial heterogeneity affects species coexistence.

### 4.1 Effects of habitat orientation on zooplankton behavior and grazing rate

Spatial storage effect is one of potential mechanisms contributing to coexistence, particularly between closely related species (Schäffler, Saborowski, & Kappeler, 2015). The spatial storage effect operates when competing species exhibit different responses to spatial variation and the strength of competition varies spatially (Abrams & Holt, 2002; Jiang & Morin, 2007). Morphological differences can be one of the important causes accounting for the storage effect as they can result in a differential locomotor pattern (Emlet, 1994) and thus in the use of space between competing species (Basset, 1995; Westphal et al., 2006). For example, *D. magna* individuals are usually found in the upper layer of the experimental vessel (Weber & Noordwijk, 2002) and move vertically more frequently compared with *M. micrura* (Pan et al., 2015). This might be attributed to higher ratio of body depth (the maximum dorsoventral depth) to body width in *D. magna* than *M. micrura* (especially between juveniles of the two grazer species), which permitted to reduce the pressure drag in vertical movement in *D. magna*. Consequently, *D. magna* individuals would be more adapted to narrow and deep habitats compared to *M. micrura*.

As expected, increasing chamber height led to deceases in average swimming velocity in both grazers species, but the decrease in magnitude was more obvious for *M. micrura* than that for *D. magna* individuals. Aquatic organisms (e.g., large zooplanktons with Reynolds numbers >1) have to spend more energy to swim upward for the same distance than individuals swimming horizontally as a result of additional costs of overcoming gravity (Garcia et al., 2007; Pan et al., 2015). Therefore, zooplankton individuals usually decrease average swimming velocity with increasing duration of upward swimming in tall spaces in order to allocate more energy to the survival and growth of grazers (Pan & Sun, 2016; Seuront, 2006; Svetlichny & Hubareva, 2005). As a result, in the present study, *D. magna* individuals should have allowed more energy and time in swimming activity in tall chambers because of a higher morphological adaptation to reduced water drag force, as evidenced by the less reduced average swimming velocity for *D. magna* compared to *M. micrura* in the tall chambers (Figure 3a,b). Because the grazing rate of zooplanktons is often positively correlated with swimming velocity (Visser & Kiørboe, 2006; Pan et al., 2015; and the present study, see Appendix 4), it is not surprising that the differential effects of habitat orientation on swimming velocity led to the significant difference in grazing rate between the two grazer species.

### 4.2 Effects of habitat orientation on interspecies competition

The altered grazing rate by spatial variation might have further transmitted to affect the development of the study grazers. Previous studies show that low grazing rate often limits body growth and induce high mortality rate in zooplankton individuals (Ashforth & Yan, 2008; Ismail, Qin, & Seuront, 2011), and zooplankton organisms often reduce reproduction rate under starvation so as to improve body reserves to avoid possible future food shortages (Bradley, Perrin, & Calow, 1991). In the present study, the lower values of body size, reproduction rate, and survival rate in *M. micrura* and/or *D. magna* in tall chambers could simply be attributed to lower grazing rate, which was evidenced by the closely relationships between average swimming velocity and life history parameters in the two grazer species (Figure 5a–f). Moreover, high phytoplankton biomass was observed in tall chambers possibly because of decreased grazing rate of grazer individuals.

**Figure 7:**
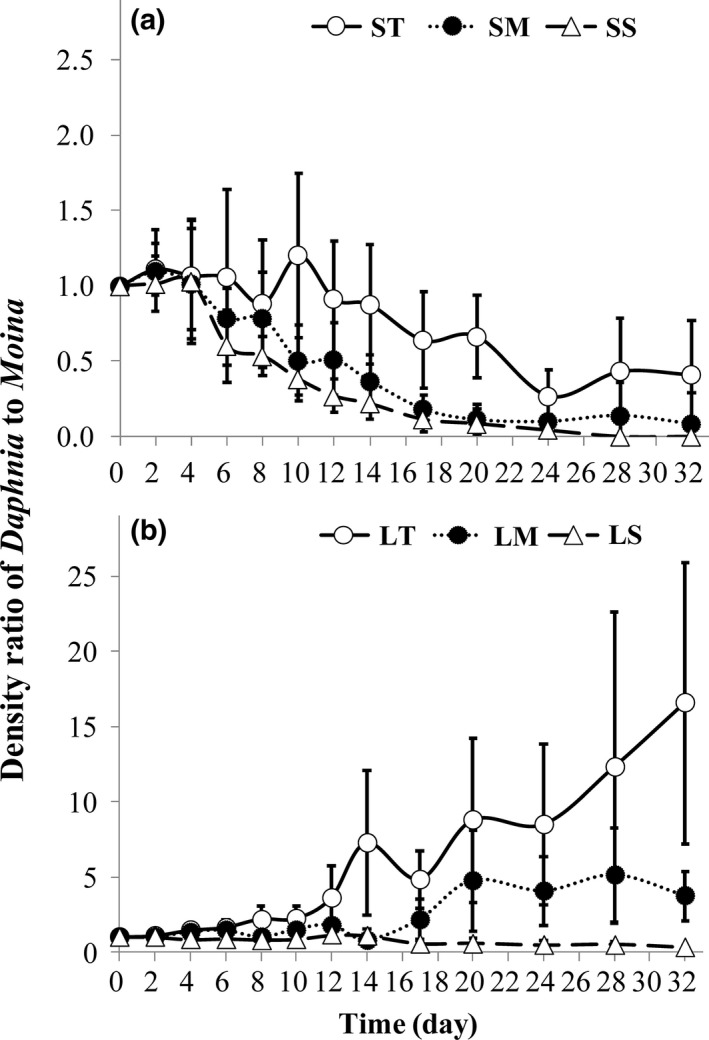
Density ratio (means ± 1 *SD*,* n* = 6) of *Daphnia magna* to *Moina micrura* in the small (a) or large (b) chamber through the month‐long experiment under three levels of habitat orientation. SS, small volume and short chamber height; SM, small volume and medium chamber height; ST, small volume and tall chamber height; LS, large volume and short chamber height; LM, large volume and medium chamber height; LT, large volume and tall chamber height

It is clear that the interspecific difference in swimming velocity and grazing rate was responsible for the observed difference in the population density between *D. magna* and *M. micrura* in the cocultured experiment. Specifically, presumably because the swimming velocity ratio of *D. magna* to *M. micrura* increased with increasing chamber height, the ratios of body size and survival rate of *D. magna* to *M. micrura* also increased with increasing chamber height (see Figure 5i,j). Consequently, the density ratio of *D. magna* to *M. micrura* gradually increased with increasing chamber height, regardless of chamber volume (Figure 7).

Additionally, we have clearly shown that chamber volume also played an important role in modulating interspecies competition. Small space can inhibit the swimming activity of zooplankton species, especially for large ones (Dodson, Ryan, Tollrian, & Lampert, 1997). Therefore, swimming velocity might be stimulated by increasing chamber volume in both grazer species, but should be more obvious in large‐sized *D. magna* than small‐sized *M. micrura*. Consistently, increasing chamber volume increased the average swimming velocity of both grazer species, but was more prominent in *D. magna* than in *M. micrura* due to a higher increase in the velocity of both upward (vertically ascent) and horizontal movements. Consequently, the grazing rate, body growth, survival and reproduction rate were more significantly improved in *D. magna* relative to *M. micrura*, resulting in a higher density ratio of *D. magna* to *M. micrura* in large chambers than small ones, regardless of chamber orientation.

### 4.3 Summary

In summary, our results provide experimental evidence that changes in habitat orientation and size affect species differentially likely because of the difference in their body morphology and body size, which further affected the outcome of interspecies competition. This finding indicates that variation in habitat orientation can serve as an additional dimension of spatial heterogeneity, permitting more species to coexist and promote species diversity within biological communities (Davies et al., 2005; Kneitel & Chase, 2004). Indeed, habitat orientation may contribute to species coexistence in nature. For instance, leopards better coexist with lions while competing for captured prey in treed African savanna than untreed grassland; vertically and horizontally flat bodied fishes coexist in tropical reefs bearing various oriented habitats that are possibly similar in both size and shape (Gardiner & Jones, 2005; Swanson, Arnold, Kosmala, Forester, & Packer, 2016). Thus, the effect of habitat orientation on species interaction should be further studied in various ecosystems to fully understand the importance of habitat orientation to community coexistence and species diversity.

## CONFLICT OF INTEREST

None declared.

## AUTHOR CONTRIBUTIONS

Ying Pan and Shucun Sun designed the research. Ying Pan and Yunshu Zhang performed all the experiments. Ying Pan analyzed the data. Ying Pan and Shucun Sun wrote the manuscript and all authors contributed comments.

## ANIMAL ISSUES

This research conducted under Law of the People's Republic of China on the Protection of Wildlife (August 28, 2004). No permits were required to carry out this study. All animal work was approved by the Animal Care Committee at Nanjing University.

## APPENDIX 1 Cell densities (means ± *SD*,* n* = 6) of *Chlorella pyrenoidosa* in treatment containing only algae cells across different layers of water column in the large volume and small‐based experimental chambers. Algal density was monitored at the 1st, 2nd, and 3rd hour in the experiment with a frequency of 5 min stirring per hour (see more details in text). Different letters indicate significant differences (*p* < .05) among treatments. Multiple comparisons of means were performed using Tukey test at *p* = .05, following one‐way ANOVAs.

### 1.1

**Table:**

		1 hr	2 hr	3 hr
Cell density (×10^−5^ cell/ml)	Bottom layer (12–16 cm)	0.254 ± 0.030a	0.248 ± 0.036a	0.274 ± 0.040a
	Middle layer (6–10 cm)	0.252 ± 0.028a	0.249 ± 0.041a	0.261 ± 0.044a
	Top layer (0–4 cm)	0.256 ± 0.044a	0.245 ± 0.039a	0.256 ± 0.039a

## APPENDIX 2 Dissolved oxygen concentration (means ± *SD*,* n* = 6) in different plankton systems under different microcosm conditions during the grazing experiment. Different letters indicate significant differences (*p* < .05) among treatments. Multiple comparisons of means were performed using Tukey test at the 0.05 significance level.

### 2.1

**Table:**

		In the absence of grazers	In the presence of both grazer species
Small volume	Short chamber height	7.55 ± 0.73a	7.51 ± 0.50a
	Medium chamber height	7.29 ± 0.37a	7.01 ± 0.53a
	Tall chamber height	7.48 ± 0.73a	6.79 ± 0.59a
Large volume	Short chamber height	7.88 ± 0.42a	7.05 ± 0.50a
	Medium chamber height	7.52 ± 0.26a	6.68 ± 0.50a
	Tall chamber height	7.62 ± 0.73a	7.35 ± 0.60a
